# Corneal Epithelial Removal with a Newly Designed Epithelial Brush

**DOI:** 10.1155/2021/4668056

**Published:** 2021-11-16

**Authors:** Ho Seok Chung, Seung Hwan Moon, Soon-Suk Kang, Minseop Kim, Hun Lee, Hungwon Tchah, Jae Yong Kim

**Affiliations:** ^1^Department of Ophthalmology, Dankook University Hospital, Dankook University College of Medicine, Cheonan 31116, Republic of Korea; ^2^Department of Ophthalmology, University of Ulsan College of Medicine, Asan Medical Center, Seoul 05505, Republic of Korea; ^3^Biomedical Research Center, Asan Institute for Life Science, Asan Medical Center, Seoul 05505, Republic of Korea; ^4^Cheonan Seoul Eye Center, Cheonan, Republic of Korea

## Abstract

This study aimed to evaluate and compare the effectiveness of a newly developed epithelial removal brush with conventional methods in a rabbit model of corneal epithelial defects. The corneal epithelia of thirty-seven rabbits were removed by three different methods including blades (blade group), newly developed epithelial brushes (Ocu group), and conventional rotating brushes (Amo group). The defect area was measured with light microscopy immediately and at 4, 18, 24, and 50 hours after removal. Corneas were obtained immediately and at 24 and 50 hours and subjected to hematoxylin and eosin (H&E) and immunofluorescence staining using proliferating cell nuclear antigen (PCNA) and phosphorylated heat shock protein 27 (pHSP27) antibodies. The residual stromal surface was observed by scanning electron microscopy (SEM). In the Ocu group, epithelia were significantly recovered at 18, 24, and 50 hours compared with immediately after removal, and in the blade and Amo groups, epithelia were significantly recovered only at 50 hours after epithelial removal. The expression levels of PCNA and pHSP27 did not differ among three groups. There was significantly more inflammatory cell infiltration in the blade group than in the other groups. SEM showed a more regular and uniform residual stromal surface in the Ocu group than in the other groups. The newly developed epithelial brush showed better polishing ability and led to earlier significant epithelial recovery and a more regular and uniform stromal surface than conventional methods in this rabbit model of epithelial defects. Accumulation of clinical data is expected to expand the scope of application of new brushes for laser surface ablation.

## 1. Introduction

Laser refractive surgery is a technique that has been widely used for approximately 40 years to correct refractive error and is performed with an excimer laser to ablate the cornea to deform the corneal structure. Laser in situ keratomileusis (LASIK) is a method of flap formation and ablation of the underlying stroma and is often associated with decreased postoperative pain and rapid visual acuity recovery, but there is a risk of flap-related complications [1, 2]. Laser surface ablation techniques including photorefractive keratectomy (PRK), laser-assisted subepithelial keratectomy (LASEK), and epi-LASIK (epithelial LASIK) have the advantage of maintaining the biomechanical strength of the cornea compared with LASIK, but they also have disadvantages such as increased time to recovery of visual acuity, subepithelial clouding, and myopic regression [3].

The first step of surface ablation is removal of the corneal epithelium. Keeping the corneal stromal surface as smooth as possible without damage is essential to prevent postoperative complications including pain and corneal haze by facilitating rapid epithelial healing [4–6]. Therefore, the use of an epithelial removal technique that leaves a smooth stromal surface is clinically important, and mechanical removal using a blunt spatula or rotating brush and removal with alcohol or an excimer laser are commonly performed [7, 8]. The rotating brush effectively removes the corneal epithelium while minimizing damage to Bowman's membrane and allows more rapid healing of epithelial defects with less postoperative haze than blunt mechanical debridement [7, 9–11].

The Occubrush® epithelial brush (Occutech, Gyeonggi-do, Korea) is a recently developed corneal epithelial brush that facilitates accurate epithelial removal due to its uniform center and curvature structure. The aim of our study was to evaluate the effectiveness and safety of this newly developed epithelial brush and compare it with the widely used rotating brush and sharp blade in a rabbit model of corneal epithelial defects.

## 2. Methods

### 2.1. Animals

Thirty-seven New Zealand white rabbits, each weighing between 2.5 and 3.0 kg, were used in this study. They were kept in standard rabbit cages with good environmental control. All experimental procedures conformed to the guidelines in the Association for Research in Vision and Ophthalmology (ARVO) Statement for the Use of Animals in Ophthalmic and Vision Research (ARVO Animal Policy). This study was conducted in strict accordance with adherence to the relevant national and international guidelines regarding animal handling as mandated by the Institutional Animal Care and Use Committee of the University of Ulsan College of Medicine. This committee reviewed and approved the animal study protocol (2019-13-247).

All interventions were performed under anesthesia, and all efforts were made to minimize suffering. All rabbits were anesthetized with an intramuscular injection of a mixture of tiletamine and zolazepam (Zoletil®50; Virbac Corp., Carros Cedex, France) and xylazine (Rompun; Bayer AG, Leverkusen, Germany). Then, topical anesthesia was given with 0.5% proparacaine hydrochloride (Alcaine®; Alcon Laboratories, Fort Worth, TX). The rabbits were randomly divided into three groups according to the epithelial removal method. The polishing ability and scanning electron microscopy findings were blindly evaluated by one practitioner (JYK) who was blinded to the group assignment. In the blade group, the corneal epithelium of approximately 6 mm from the periphery to the center was quickly and gently removed by mechanical debridement using a sharp scalpel blade (#15, Kiato plus blade, MDSS GmbH, Hannover, Germany), and the removed site was washed with normal saline. In the Ocu and Amo groups, the corneal epithelium was removed at room temperature (RT) using the newly developed epithelial brush (Occubrush, product photo is attached in Supplementary Materials) and a rotating brush (Amoils epithelial scrubber; Innovative Excimer Solutions, Inc., Toronto, Canada), respectively. Removal of the corneal epithelium was performed for approximately 10 seconds, and the diameter of the removed epithelium was approximately 6 mm.

### 2.2. Comparison of Polishing Ability and Wound Healing in a Rabbit Model

Thirty-six rabbits were divided into the three groups mentioned above with twelve rabbits (24 eyes) per group to compare the polishing ability of each technique and wound healing in each group. One untreated rabbit was included as a control. To compare the polishing ability of each technique, the corneal epithelium was observed with a light microscope immediately after removal. After epithelial removal, wound healing was observed with a light microscope under cobalt blue light following instillation of 2% sodium fluorescein (Bausch and Lomb, Inc., Rochester, NY), and photographs were taken immediately after epithelial removal and at 4, 18, 24, and 50 hours after epithelial removal. The area of the epithelial defect in the photographs taken was calculated using ImageJ software (version 1.62f; available at https://rsbweb.nih.gov/ij/; developed by Wayne Rasband, National Institutes of Health, Bethesda, MD).

### 2.3. Immunofluorescence and Hematoxylin and Eosin (H&E) Staining

Three rabbits in each group were sacrificed immediately and 24 and 50 hours after removal. Both eyes were enucleated and fixed for 24 hours in neutral-buffered formalin (3.7% formaldehyde). The cornea was obtained from each eye by making a stab incision through the pars plana and cutting circumferentially with scissors. The separated cornea was embedded into a paraffin block, and the processed tissue was sectioned into 4 *μ*m thick sections and mounted on slides. After deparaffinization, the slides were heated in 0.01 M sodium citrate buffer solution (pH 6.0) at 90–100°C for 30 minutes for antigen retrieval. The tissues were then blocked with 0.1% bovine serum albumin (BSA) and 5% donkey serum (Jackson ImmunoResearch Laboratories Inc., West Grove, PA) at RT for 30 minutes. The slides were washed three times for 10 minutes each and incubated with primary antibodies for proliferating cell nuclear antigen (PCNA; 1:200, NB500-106; Novus Biologicals, Inc., Centennial, CO) and phosphorylated heat shock protein 27 (pHSP27; 1:200, ab5581; Abcam, Inc., Cambridge, MA) overnight at 4°C. They were incubated with the secondary antibodies (1:1000) at RT for 1 hour. The slides were washed three times for 10 minutes each and stained with 4′-6-diamidino-2-phenylindole (DAPI) (Vector Laboratories, Inc., Burlingame, CA) for 5 minutes to counterstain the cell nuclei. After dehydration, the slides were mounted in fluorescence mounting medium and examined using an LSM780 confocal microscope (Carl Zeiss Meditec AG, Jena, Germany). The remaining sections were subsequently used to confirm the infiltration of inflammatory cells in the cornea by H&E staining.

### 2.4. Scanning Electron Microscopy (SEM)

A total of nine rabbits with three rabbits per group were assigned to observe the residual stromal surface using SEM. All rabbits were sacrificed immediately after epithelial removal, all right eyes were enucleated, and the anterior segment including the cornea was obtained from each eye. The separated cornea was prefixed with 1% paraformaldehyde and 1% glutaraldehyde in 0.1 M cacodylate buffer (pH 7.4) for 24 hours at 4°C, postfixed with 2% osmium tetroxide in 0.1 M cacodylate buffer at RT, and dehydrated with progressive concentrations of ethanol. The dehydrated sample was replaced with ethanol and isoamylacetate, and the tissue sample substituted with pure isoamylacetate was once again dried with a critical point dryer. The sample was coated with platinum (Au) using an ion coater and examined with a scanning electron microscope (S-4500; Hitachi, Inc., Tokyo, Japan).

### 2.5. Statistical Analysis

The Wilcoxon signed-rank test was used to compare the area of the epithelial defect according to the time after epithelial removal in each group. The Kruskal–Wallis test was used to compare the proportion of the epithelial defect area immediately after epithelial removal and at each time point between groups. Statistical significance was set at *P* < 0.05. All statistical analyses were performed using SPSS version 21.0 software (IBM SPSS Inc., Chicago, IL).

## 3. Results

### 3.1. Polishing Ability

**Figure 1:**
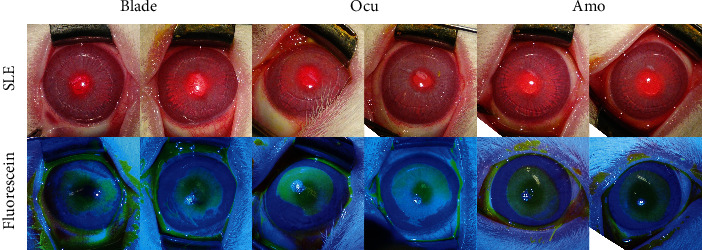
Light microscopic images taken immediately after epithelial removal before and after fluorescence staining in the three groups.

Light microscopic images taken immediately after epithelial removal and under cobalt blue light after fluorescence staining showed irregular and rough borders of the epithelial defects in the blade group (Figure 1). However, in the Ocu and Amo groups, 50 *μ*m or more of the corneal epithelial layer was completely removed, revealing excellent polishing ability of these methods. The Ocu group showed regular and clear borders of the epithelial defects, and the epithelium was removed in a precise circular shape. The Amo group also showed clean borders of the epithelial defects, but the epithelium was removed in a more oval than circular shape.

### 3.2. Epithelial Wound Healing

The ratio of the epithelial defect area at each time point (4, 18, 24, and 50 hours) after epithelial removal to the epithelial defect area immediately after epithelial removal was calculated (Table 1). In the blade and Amo groups, the ratio significantly decreased only at 50 hours after epithelial removal. However, in the Ocu group, the ratio significantly decreased at 18, 24, and 50 hours (all *P* < 0.05). There were no significant differences among the three groups at 4, 18, and 24 hours. At 50 hours, only Ocu and Amo groups exhibited complete epithelial healing.

### 3.3. Immunofluorescence for PCNA and pHSP27

**Figure 2:**
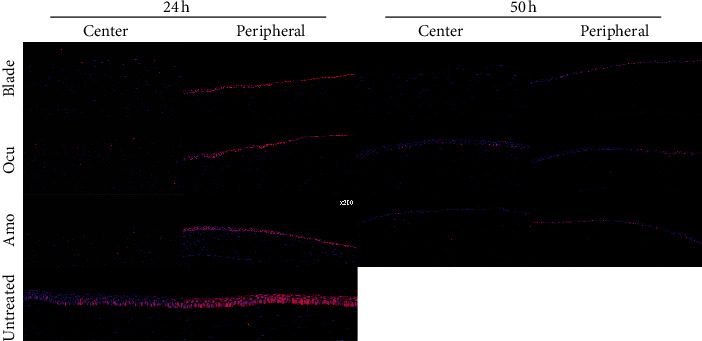
Proliferating cell nuclear antigen (PCNA) staining at 24 and 50 hours after epithelial removal in the three groups and an untreated control.

PCNA staining was performed at 24 and 50 hours after epithelial removal in the blade, Ocu, and Amo groups and in untreated controls (Figure 2). In untreated controls, there was more PCNA expression in the peripheral cornea close to the limbus where stem cells were located than in the central cornea. At 24 hours, PCNA was not expressed in the central cornea in three groups, indicating that the central corneal epithelia had not yet recovered. However, there was increased PCNA expression in the peripheral cornea where wound healing had occurred in three groups, and there was no difference in the expression levels among the three groups. At 50 hours after epithelial removal in the blade group, the epithelial defect remained in the central cornea; however, in the Ocu and Amo groups, the epithelia were completely healed, and PCNA was expressed in the central cornea, with no significantly different expression between the two groups. There was no difference in the PCNA expression among the three groups in the peripheral cornea at 50 hours.

**Figure 3:**
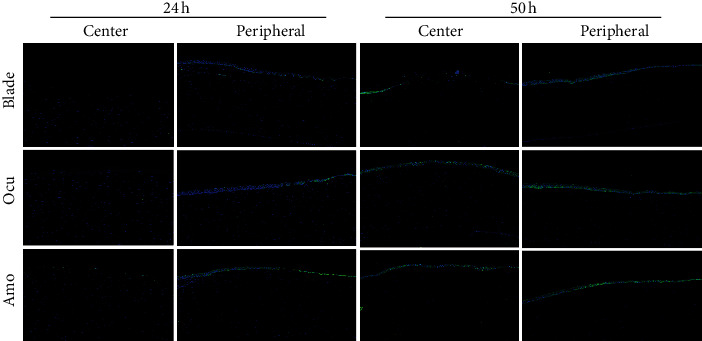
Phosphorylated heat shock protein 27 (pHSP27) staining at 24 and 50 hours after epithelial removal in the three groups.

At 24 and 50 hours after epithelial removal in three groups, pHSP27 expression was investigated (Figure 3). At 24 hours, pHSP27 was not expressed in the central cornea, indicating that the central corneal epithelia had not yet recovered. However, pHSP27 was expressed in the peripheral cornea where wound healing had occurred in the three groups, and there was no difference in the expression levels among three groups. At 50 hours after epithelial removal in the blade group, the epithelial defect remained in the central cornea; however, in the Ocu and Amo groups, the epithelia were completely healed, and pHSP27 expression was found in the central cornea, with no significantly different expression between the two groups. There was no difference in pHSP27 expression among the three groups in the peripheral cornea at 50 hours.

### 3.4. H&E Staining

**Figure 4:**
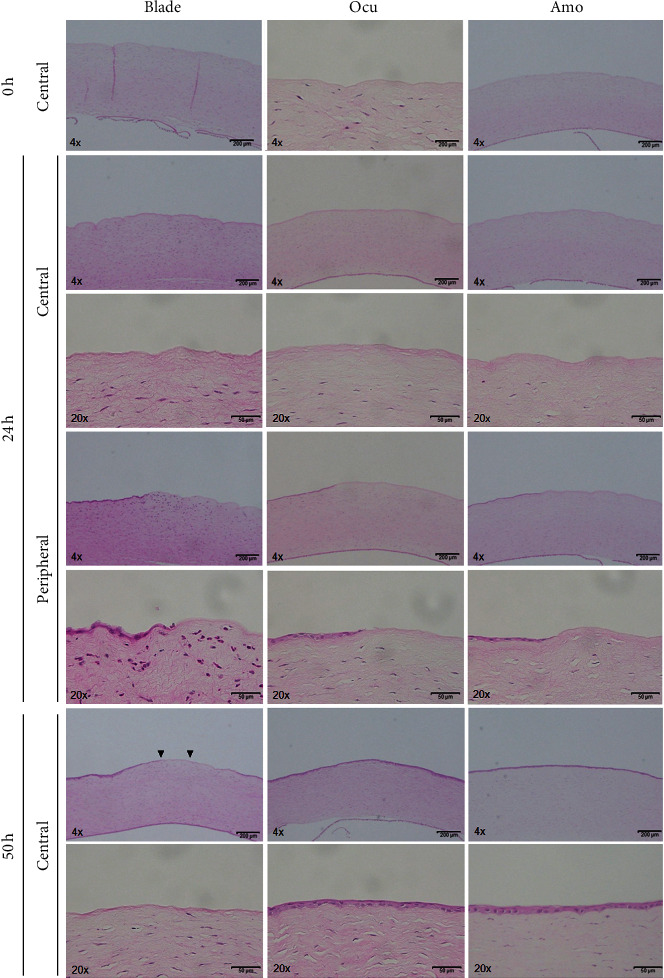
Hematoxylin and eosin staining immediately and at 24 and 50 hours after epithelial removal in the three groups.

H&E staining was performed in the three groups (Figure 4). Corneal epithelia were not present in the central cornea immediately after epithelial removal in all groups. At 24 hours, no inflammatory cell infiltration was observed in the central cornea in all groups, but the blade group had significantly more infiltration in the peripheral cornea where wound healing had occurred than the Ocu and Amo groups (*P* < 0.05). At 50 hours, the epithelial defect remained in the central cornea in the blade group, but in the Ocu and Amo groups, the epithelial defects had completely healed. No inflammatory cell infiltration was observed in all groups at 50 hours.

### 3.5. SEM

**Figure 5:**
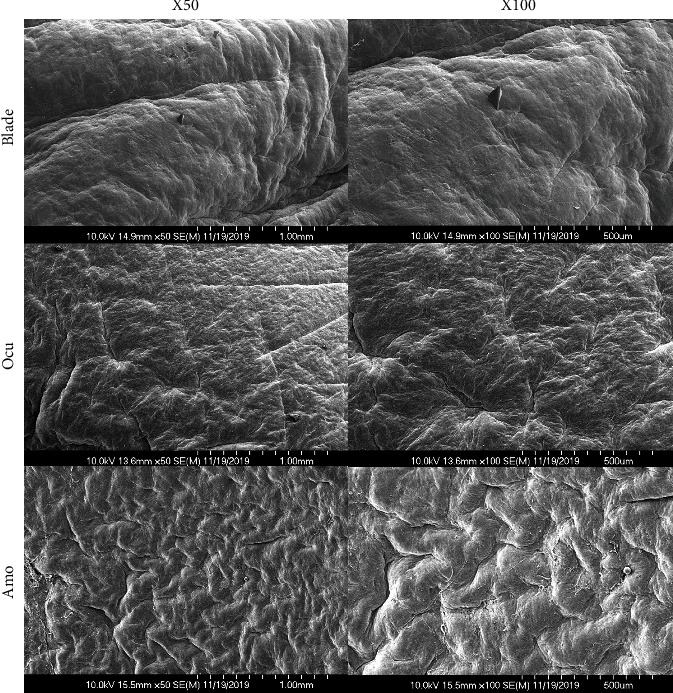
Residual stromal surfaces investigated by scanning electron microscopy (SEM) in the three groups (50x and 100x magnification).

SEM images at 50x and 100x magnification were obtained in the three groups (Figure 5). In the blade group, several grooves measuring from 10 to 20 *μ*m were observed, and the residual stromal surface was rougher and more irregular than in the other groups. However, in the Ocu and Amo groups, flat, regular, and uniform residual stromal surfaces were found. There was more regularity of the residual stromal surface in the Ocu group than in the Amo group.

## 4. Discussion

The newly developed epithelial brush was designed to be different from the conventional brush in its microscopic structure to increase the accuracy of epithelial removal. In the manufacturing process, a jig suitable for the corneal curvature was used to reduce decentralization and to adhere to the cornea at a constant pressure when removing the epithelium. In addition, the noncontact processing method using vibrations and air can reduce the occurrence of foreign bodies on the brush surface.

In this study, three different methods of epithelial removal for laser surface ablation were evaluated. When the newly developed epithelial brush was used, the epithelium was removed with regular, circular, and clean margins. This method led to earlier significant recovery and a smoother surface than the other epithelial removal methods. Overall, the Ocu group showed better results than the blade group and showed similar or better results than the Amo group. In the Ocu and Amo groups, staining for PCNA and pHSP27, indicators of epithelial proliferation and migration, occurred only in the peripheral cornea at 24 hours and in the central cornea at 50 hours. Furthermore, less inflammatory cell infiltration was exhibited in these groups than the Blade group. To the best of our knowledge, only comparative studies of clinical outcomes according to the epithelial removal method and studies of changes at the cellular level after general PRK have been conducted [4, 12–16]. This study is meaningful in that the differences in wound healing at the cellular level were compared according to the epithelial removal method.

When the polishing ability was compared immediately after epithelial removal, as expected based on the results of a previous clinical study, the most irregular and unclear margins of the epithelial defect were exhibited in the blade group [7]. The Ocu and Amo groups exhibited sufficiently regular and clean margins, but the Ocu group exhibited a rounder shape of the removed epithelium than the other groups. The Amoils rotating brush consists of a disposable circular brush and a handle with a motor that rotates it, which may lead to an oval shape of the removed epithelium due to fine movement and irregular contact of the rotating head [17]. Although the area was not large, unintended areas of the epithelium could be removed in the Amo group. The newly developed epithelial brush used a jig suitable for the corneal curvature to prevent decentralization during the manufacturing process. As a result, the new brush was uniformly adhered to the ocular surface, and the corneal epithelium was precisely removed in a circular shape.

In previous studies that observed the corneal surface with SEM after epithelial removal, the residual stromal surface after epithelial removal with a brush was smoother without grooves than after mechanical removal with a spatula, and there were few remaining epithelial cells [7, 18]. Similarly, in this study, the residual stromal surfaces on SEM images of the blade and Amo groups were comparable with previous results using a sharp blade and rotating brush, respectively [18]. The newly developed epithelial brush uses a noncontact processing method using vibrations and air to reduce the occurrence of foreign bodies on the brush surface. The Ocu group exhibited an overall uniform residual stromal surface due to decreased foreign body generation and to the structural suitability of the newly developed epithelial brush.

When comparing the epithelial defect area over time, the ratio of epithelial defects significantly decreased at 18 hours after removal in the Ocu group, but significantly decreased at 50 hours in the other groups. This result can be attributed to the more uniform and flatter residual surface in the Ocu group than in the Amo group as revealed with SEM as well as to the superior polishing ability in the Ocu group. However, there was no significant difference between the Amo and Ocu group at any time point, and this result needs to be confirmed by comparing wound healing after laser refractive surgery in a clinical setting. In a previous clinical study, complete healing was observed in 64% of patients treated using a brush 3 days after PRK and in 36% of patients treated using a blunt scraper [7]. The postoperative uncorrected visual acuity was also better, and corneal haze occurred less frequently in the brush group than in the scraped group [7]. In a relatively recent clinical study, complete healing was observed in both the brush and crescent knife groups during 5 days after PRK, and the results of the present study are comparable with those of this previous study [15].

PCNA is naturally expressed in proliferating cells, and pHSP27 is involved in epithelial migration and apoptosis [19–21]. Both PCNA and pHSP27 were expressed only in the peripheral cornea in all groups at 24 hours after epithelial removal and in the central cornea in the Ocu and Amo groups at 50 hours but only in the peripheral cornea in the blade group at this time point. In experiment on the wound healing process after refractive surgery, proliferation and migration of residual keratocytes after apoptosis begin from 12 to 24 hours and markedly diminish approximately after 1 week [4, 22]. Our results at 24 and 50 hours are consistent with those from previous reports, and in the blade group, the wound healing process may have been slower than in the other groups due to irregular wound margins and residual stromal surface.

Inflammatory cell infiltration begins from 8 to 12 hours after injury and penetrates through the broken blood-aqueous barrier [4]. After phagocytosis of apoptotic cell bodies and other residual cell fragments, these cells disappear after epithelial closure [14]. Other previous study reported that mechanical removal upregulates the expression of inflammatory cytokines compared to ethanol removal [23]. Our H&E staining results are consistent with these reports. In the blade group, significantly more inflammatory cell infiltration was observed around the margins than in other groups; this finding may be related to the poor polishing ability of this technique. In the Ocu and Amo groups, after wound healing was completed at 50 hours after removal, no inflammatory cells were observed.

Using the conventional brush, the uncorrected distant visual acuity showed 20/20 or more in more than 95% of cases 12 months after PRK, and a difference of 0.12 diopters from the intended spherical equivalent which was satisfactory [24, 25]. Even though our findings showed the newly developed brush had less inflammation and more regular epithelial defects, post-LASEK refractive outcomes might be quite unrelated to these factors. Further clinical trials need to be carried out to determine whether the newly developed brush can provide additional improvement in visual acuity and refractive outcomes compared to the conventional brush through the uniform residual stromal surface and rapid epithelial recovery.

## 5. Conclusion

In conclusion, due to its structural originality and specificity, the newly developed epithelial brush showed better polishing ability and led to earlier significant epithelial recovery and a more regular and uniform residual stromal surface than the conventional rotating brush in this rabbit model of epithelial defects. We also observed the wound healing process at the cellular level according to the epithelial removal method. Our study is meaningful in accumulating data for clinical research, and it is necessary to confirm the clinical relevance of this experimental results through clinical research. In addition, clinical studies comparing the refractive outcomes and intraoperative or postoperative pain associated with this new method with those of other epithelial removal methods are expected to expand the scope of application.

## Figures and Tables

**Table 1:** Ratio of the epithelial defect area at each time point after epithelial removal to the epithelial defect area immediately after epithelial removal.

Time	Blade group	Ocu group	Amo group	*P* value^†^
4 hours	0.99 ± 0.06	1.02 ± 0.05	0.93 ± 0.10	0.30
18 hours	0.88 ± 0.08	0.86 ± 0.05^*∗*^	0.79 ± 0.14	0.58
24 hours	0.76 ± 0.11	0.65 ± 0.04^*∗*^	0.71 ± 0.08	0.28
50 hours	0.09 ± 0.05^*∗*^	Completely	Completely	NA^‡^
		Healed	Healed	

^*∗*^ Statistically significant difference in the epithelial defect area between immediately after epithelial removal and at each time point. ^†^ Kruskal–Wallis test. ^‡^*P* value could not be obtained due to the complete healing of the epithelial defect in the Ocu and Amo groups.

## Data Availability

The data used to support the findings of this study are available from the corresponding author upon request.

## Supplementary Materials

Supplementary Materials Supplementary Figure 1. A product photo of the newly developed epithelial brush (Occubrush).
