# Supplementary figures and images for: Corneal Epithelial Removal with a Newly Designed Epithelial Brush
Source: J Ophthalmol. 2021 Nov 16;2021:4668056. doi: 10.1155/2021/4668056 (PMC8828349; doi:10.1155/2021/4668056)

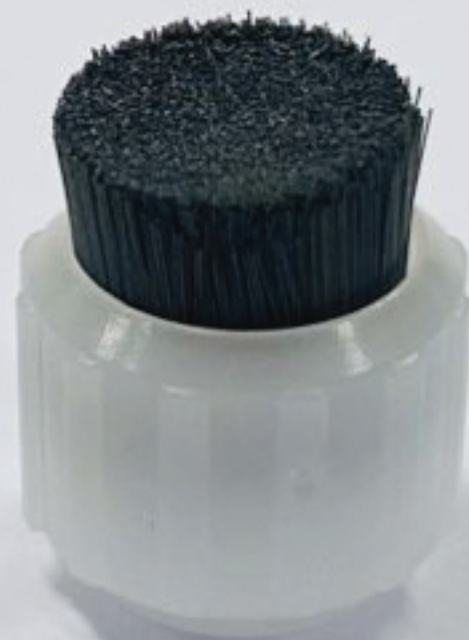

Supplement: Supplementary Materials — Supplementary Figure 1. A product photo of the newly developed epithelial brush (Occubrush). [file 4668056.f1.pdf]
